# Supplementary material for: Uncertainty in lung cancer stage for survival estimation via set‐valued classification
Source: Stat Med. 2022 Jun 8;41(19):3772–88. doi: 10.1002/sim.9448 (PMC9540678; doi:10.1002/sim.9448)
Supplement: Supplementary file 1 — Data S1: Supporting information [file SIM-41-3772-s001.pdf]

APPENDIX

A CLASSIFICATION MEASURES

TABLE A1 Classification performance measures.

Measure	Definition	
	Class-Specific	Macro-Average
Accuracy	$\sum_{i=1}^{K} \frac{tp_i+tn_i}{tp_i+tn_i+fp_i+fn_i}$	$\sum_{i=1}^{K} \frac{tp_i+tn_i}{tp_i+tn_i+fp_i+fn_i}/K$
Sensitivity (Recall)	$\sum_{i=1}^{K} \frac{tp_i}{tp_i+fn_i}$	$\sum_{i=1}^{K} \frac{tp_i}{tp_i+fn_i}/K$
Specificity	$\sum_{i=1}^{K} \frac{tn_i}{tn_i+fp_i}$	$\sum_{i=1}^{K} \frac{tn_i}{tn_i+fp_i}/K$
Positive Predictive Value (Precision)	$\sum_{i=1}^{K} \frac{tp_i}{tp_i+fp_i}$	$\sum_{i=1}^{K} \frac{tp_i}{tp_i+fp_i}/K$

Notes: tp_i denotes observation i as true positive, tn_i true negative,fp_i false positive, fn_i false negative, K is the number of class labels.

B SIMULATION STUDY

Data are simulated using R 3.6.1 using a Mersenne-Twister random number generator with an input seed "33." The outcome Y_i is based on a multinomial logit where the multinomial probabilities are calculated as follows:

$$p_{ik} = \begin{cases} \frac{\exp(X_i'b_1)}{1+\exp(X_i'b_1)+\exp(X_i'b_2)} & \text{for } k = 1 \\ \frac{\exp(X_i'b_2)}{1+\exp(X_i'b_1)+\exp(X_i'b_2)} & \text{for } k = 2 \\ \frac{1}{1+\exp(X_i'b_1)+\exp(X_i'b_2)} & \text{for } k = 3, \end{cases}$$

where X_i is a vector of predictor values for observation i and b_1 and b_2 are vectors of coefficients corresponding to classes 1 and 2, respectively. We set the coefficients:

$$X_i'b_1 = 1.8 \times (-8.25 + 0.2X1_i + 0.24(X7_i \times X10_i) - 0.3X3_i + 0.21\sqrt{X14_i} - 0.9X9_i + 0.9X11_i + 0.1\sin(X5_i))$$

$$X_i'b_2 = 1.8 \times (-1.95 + 0.04X1_i + 0.5(X7_i \times X10_i) - 0.03X3_i + 0.032\sqrt{X14_i} - 0.02X9_i + 0.003X11_i + 0.31\sin(X5_i))$$

TABLE B2 Simulation covariates.

			Used for Prediction		
**Covariate**	**Distribution**	**Used to Generate Y_i**	**Scenario 1**	**Scenario 2**	**Scenario 3**
X1	N(75,5)	×	×	×	
X2	N(45000,10000)			×	×
X3	N(23,4)	×	×	×	
X4	N(70,5)			×	×
X5	N(5,2)	×	×	×	×
X6	N(0,1) ‡		×	×	×
X7	Bernoulli(0.5)	×	×		×
X8	Bernoulli(0.25)		×		×
X9	Bernoulli(0.3)	×	×		×
X10	Bernoulli(0.7)	×	×		×
X11	Bernoulli(0.6)	×	×		
X12	Bernoulli(0.7)				×
X13	Bernoulli(0.4)		×	×	×
X14	Pois(3)†	×			×
X15	Pois(3)†				×

†X14 and X15 count variables are correlated and based on a multivariate normal distribution: MVN($\mu = (1,3), \Sigma$), where $\Sigma = \begin{pmatrix} 1 & .7 \\ .7 & 1 \end{pmatrix}$

‡X6 is the covariate used to generate survival times.

B.1 Simulation results

TABLE B3 Weighted labeling thresholds.

Scenario	Class 1	Class 2	Class 3
1	0.659	0.393	0.112
2	0.290	0.321	0.102
3	0.242	0.322	0.063

TABLE B4 Classification accuracy.

Method	Average	Class 1	Class 2	Class 3
Scenario 1				
Naive standard practice	0.89	0.95	0.84	0.89
Naive bootstrap	0.89	0.95	0.84	0.89
	(0.88, 0.91)	(0.94, 0.96)	(0.82, 0.86)	(0.87, 0.91)
Weighted bootstrap	0.86	0.94	0.80	0.84
	(0.85, 0.88)	(0.93, 0.96)	(0.77, 0.82)	(0.82, 0.86)
Scenario 2				
Naive standard practice	0.82	0.86	0.73	0.87
Naive bootstrap	0.82	0.86	0.73	0.87
	(0.80, 0.84)	(0.83, 0.88)	(0.71, 0.76)	(0.85, 0.89)
Weighted bootstrap	0.77	0.84	0.66	0.80
	(0.75, 0.79)	(0.82, 0.87)	(0.63, 0.69)	(0.78, 0.82)
Scenario 3				
Naive standard practice	0.67	0.58	0.57	0.87
Naive bootstrap	0.67	0.58	0.57	0.87
	(0.65, 0.69)	(0.55, 0.61)	(0.54, 0.60)	(0.85, 0.89)
Weighted bootstrap	0.57	0.56	0.53	0.63
	(0.55, 0.59)	(0.53, 0.59)	(0.50, 0.56)	(0.60, 0.66)

TABLE B5 Classification sensitivty.

Method	Average	Class 1	Class 2	Class 3
Scenario 1				
Naive standard practice	0.74	0.94	0.87	0.41
Naive bootstrap	0.74	0.94	0.87	0.41
	(0.71, 0.77)	(0.91, 0.96)	(0.84, 0.90)	(0.32, 0.49)
Weighted bootstrap	0.75	0.91	0.75	0.61
	(0.72, 0.79)	(0.88, 0.93)	(0.71, 0.78)	(0.53, 0.69)
Scenario 2				
Naive standard practice	0.61	0.82	0.80	0.21
Naive bootstrap	0.61	0.82	0.80	0.21
	(0.58, 0.64)	(0.78, 0.85)	(0.76, 0.83)	(0.14, 0.28)
Weighted bootstrap	0.62	0.81	0.56	0.50
	(0.59, 0.66)	(0.77, 0.85)	(0.52, 0.61)	(0.41, 0.58)
Scenario 3				
Naive standard practice	0.39	0.54	0.62	0.00
Naive bootstrap	0.39	0.54	0.62	0.00
	(0.37, 0.41)	(0.49, 0.58)	(0.58, 0.67)	(0.00, 0.01)
Weighted bootstrap	0.36	0.35	0.37	0.36
	(0.32, 0.39)	(0.30, 0.40)	(0.32, 0.41)	(0.27, 0.44)

TABLE B6 Classification specificity.

Method	Average	Class 1	Class 2	Class 3
Scenario 1				
Naive standard practice	0.91	0.96	0.81	0.96
Naive bootstrap	0.91	0.96	0.81	0.96
	(0.90, 0.92)	(0.94, 0.97)	(0.78, 0.84)	(0.95, 0.97)
Weighted bootstrap	0.90	0.97	0.85	0.88
	(0.88, 0.91)	(0.95, 0.98)	(0.82, 0.88)	(0.86, 0.90)
Scenario 2				
Naive standard practice	0.84	0.88	0.67	0.97
Naive bootstrap	0.84	0.88	0.67	0.97
	(0.83, 0.86)	(0.86, 0.91)	(0.63, 0.71)	(0.96, 0.98)
Weighted bootstrap	0.82	0.87	0.75	0.84
	(0.80, 0.84)	(0.84, 0.89)	(0.71, 0.79)	(0.82, 0.87)
Scenario 3				
Naive standard practice	0.71	0.61	0.52	1.00
Naive bootstrap	0.71	0.61	0.52	1.00
	(0.69, 0.72)	(0.57, 0.64)	(0.48, 0.56)	(1.00, 1.00)
Weighted bootstrap	0.68	0.70	0.68	0.67
	(0.67, 0.70)	(0.66, 0.73)	(0.64, 0.72)	(0.64, 0.70)

TABLE B7 Classification positive predictive value.

Method	Average	Class 1	Class 2	Class 3
Scenario 1				
Naive standard practice	0.78	0.93	0.81	0.61
Naive bootstrap	0.78	0.93	0.81	0.61
	(0.75, 0.82)	(0.91, 0.96)	(0.77, 0.84)	(0.51, 0.71)
Weighted bootstrap	0.73	0.95	0.81	0.42
	(0.70, 0.76)	(0.93, 0.97)	(0.78, 0.85)	(0.35, 0.50)
Scenario 2				
Naive standard practice	0.68	0.82	0.69	0.52
Naive bootstrap	0.68	0.82	0.69	0.52
	(0.63, 0.73)	(0.78, 0.86)	(0.65, 0.73)	(0.38, 0.66)
Weighted bootstrap	0.60	0.80	0.67	0.32
	(0.57, 0.63)	(0.76, 0.84)	(0.62, 0.72)	(0.26, 0.39)
Scenario 3				
Naive standard practice	0.40	0.47	0.54	0.20
Naive bootstrap	0.40	0.47	0.54	0.20
	(0.35, 0.47)	(0.43, 0.52)	(0.50, 0.58)	(0.08, 0.37)
Weighted bootstrap	0.36	0.43	0.50	0.14
	(0.33, 0.39)	(0.38, 0.49)	(0.45, 0.56)	(0.10, 0.17)

TABLE B8 True positive counts.

Method	Class 1	Class 2	Class 3
Scenario 1			
Naive standard practice	374	414	52
Naive bootstrap	374	414	52
	(345, 404)	(383, 444)	(39, 67)
Weighted bootstrap	362	353	78
	(332, 391)	(324, 383)	(62, 95)
Scenario 2			
Naive standard practice	326	379	27
Naive bootstrap	326	379	27
	(297, 355)	(349, 409)	(17, 37)
Weighted bootstrap	322	267	64
	(293, 351)	(240, 294)	(49, 79)
Scenario 3			
Naive standard practice	214	295	<1
Naive bootstrap	214	295	<1
	(189, 239)	(267, 323)	(0, 1)
Weighted bootstrap	140	173	46
	(119, 161)	(150, 197)	(33, 59)

TABLE B9 True negative counts.

Method	Class 1	Class 2	Class 3
Scenario 1			
Naive standard practice	575	427	838
Naive bootstrap	575	427	838
	(544, 605)	(397, 458)	(816, 861)
Weighted bootstrap	583	446	765
	(552, 613)	(415, 476)	(738, 790)
Scenario 2			
Naive standard practice	530	354	847
Naive bootstrap	530	354	847
	(500, 561)	(324, 383)	(825, 869)
Weighted bootstrap	522	394	736
	(491, 552)	(365, 425)	(709, 763)
Scenario 3			
Naive standard practice	365	274	870
Naive bootstrap	365	275	870
	(336, 395)	(247, 302)	(849, 890)
Weighted bootstrap	419	357	583
	(388, 449)	(328, 386)	(553, 613)

TABLE B10 False positive counts.

Method	Class 1	Class 2	Class 3
Scenario 1			
Naive standard practice	27	99	34
Naive bootstrap	27	99	34
	(17, 37)	(82, 118)	(23, 45)
Weighted bootstrap	19	81	107
	(11, 28)	(64, 98)	(89, 127)
Scenario 2			
Naive standard practice	71	173	25
Naive bootstrap	71	173	25
	(56, 87)	(150, 196)	(16, 35)
Weighted bootstrap	80	132	136
	(64, 97)	(111, 153)	(115, 157)
Scenario 3			
Naive standard practice	236	253	2
Naive bootstrap	236	253	2
	(211, 262)	(226, 280)	(0, 3)
Weighted bootstrap	182	170	289
	(159, 206)	(148, 194)	(261, 317)

TABLE B11 False negative counts.

Method	Class 1	Class 2	Class 3
Scenario 1			
Naive standard practice	24	60	76
Naive bootstrap	24	60	76
	(15, 34)	(46, 75)	(60, 92)
Weighted bootstrap	37	120	50
	(26, 49)	(100, 140)	(37, 64)
Scenario 2			
Naive standard practice	72	95	101
Naive bootstrap	72	95	101
	(57, 89)	(77, 113)	(83, 120)
Weighted bootstrap	76	207	64
	(61, 93)	(182, 232)	(50, 80)
Scenario 3			
Naive standard practice	185	178	128
Naive bootstrap	185	178	128
	(161, 209)	(155, 202)	(108, 149)
Weighted bootstrap	259	300	83
	(232, 286)	(272, 328)	(66, 100)

TABLE B12 Simulation study median survival days bias.

Method	Class 1	Class 2	Class 3
Scenario 1			
Naive standard practice	4	-46	18
	(-1, 10)	(-67, -28)	(5, 40)
Naive bootstrap	3	-46	19
	(-1, 10)	(-69, -27)	(6, 41)
Weighted bootstrap	3	-41	40
	(-2, 9)	(-65, -20)	(22, 63)
Scenario 2			
Naive standard practice	10	-65	30
	(2, 21)	(-88, -44)	(6, 81)
Naive bootstrap	10	-65	33
	(2, 20)	(-90, -43)	(8, 79)
Weighted bootstrap	11	-63	58
	(2, 22)	(-91, -38)	(36, 87)
Scenario 3			
Naive standard practice	13	-71	77
	(-8, 42)	(-104, -39)	(-12, 296)
Naive bootstrap	13	-72	78
	(0, 28)	(-99, -48)	(27, 146)
Weighted bootstrap	17	-76	64
	(1, 36)	(-106, -48)	(46, 84)

TABLE B13 Simulation study 90-day survival probability bias.

Method	Class 1	Class 2	Class 3
Scenario 1			
Naive standard practice	0.02	-0.09	0.21
	(0.00, 0.04)	(-0.12, -0.07)	(0.12, 0.31)
Naive bootstrap	0.02	-0.09	0.21
	(0.00, 0.03)	(-0.12, -0.07)	(0.12, 0.31)
Weighted bootstrap	0.01	-0.08	0.31
	(-0.01, 0.03)	(-0.11, -0.05)	(0.24, 0.38)
Scenario 2			
Naive standard practice	0.04	-0.13	0.26
	(0.01, 0.08)	(-0.17, -0.10)	(0.12, 0.42)
Naive bootstrap	0.04	-0.13	0.26
	(0.01, 0.07)	(-0.17, -0.10)	(0.14, 0.40)
Weighted bootstrap	0.05	-0.13	0.37
	(0.02, 0.08)	(-0.17, -0.09)	(0.29, 0.44)
Scenario 3			
Naive standard practice	0.06	-0.15	0.36
	(-0.02, 0.15)	(-0.22, -0.08)	(-0.12, 0.95)
Naive bootstrap	0.06	-0.15	0.36
	(0.01, 0.11)	(-0.19, -0.11)	(0.14, 0.58)
Weighted bootstrap	0.07	-0.16	0.38
	(0.02, 0.13)	(-0.22, -0.11)	(0.31, 0.44)

TABLE B14 Simulation study 365-day survival probability bias.

Method	Class 1	Class 2	Class 3
Scenario 1			
Naive standard practice	0.01	-0.05	0.11
	(0.00, 0.03)	(-0.07, -0.03)	(0.04, 0.18)
Naive bootstrap	0.01	-0.05	0.11
	(0.00, 0.03)	(-0.07, -0.03)	(0.05, 0.18)
Weighted bootstrap	0.01	-0.05	0.15
	(0.00, 0.02)	(-0.07, -0.02)	(0.10, 0.21)
Scenario 2			
Naive standard practice	0.03	-0.08	0.14
	(0.01, 0.06)	(-0.10, -0.05)	(0.03, 0.26)
Naive bootstrap	0.03	-0.07	0.14
	(0.01, 0.06)	(-0.10, -0.05)	(0.05, 0.24)
Weighted bootstrap	0.04	-0.08	0.18
	(0.01, 0.06)	(-0.11, -0.04)	(0.13, 0.24)
Scenario 3			
Naive standard practice	0.06	-0.10	0.19
	(-0.02, 0.15)	(-0.22, -0.08)	(-0.12, 0.95)
Naive bootstrap	0.06	-0.10	0.19
	(0.03, 0.10)	(-0.13, -0.07)	(0.08, 0.33)
Weighted bootstrap	0.07	-0.10	0.16
	(0.03, 0.11)	(-0.14, -0.06)	(0.12, 0.21)

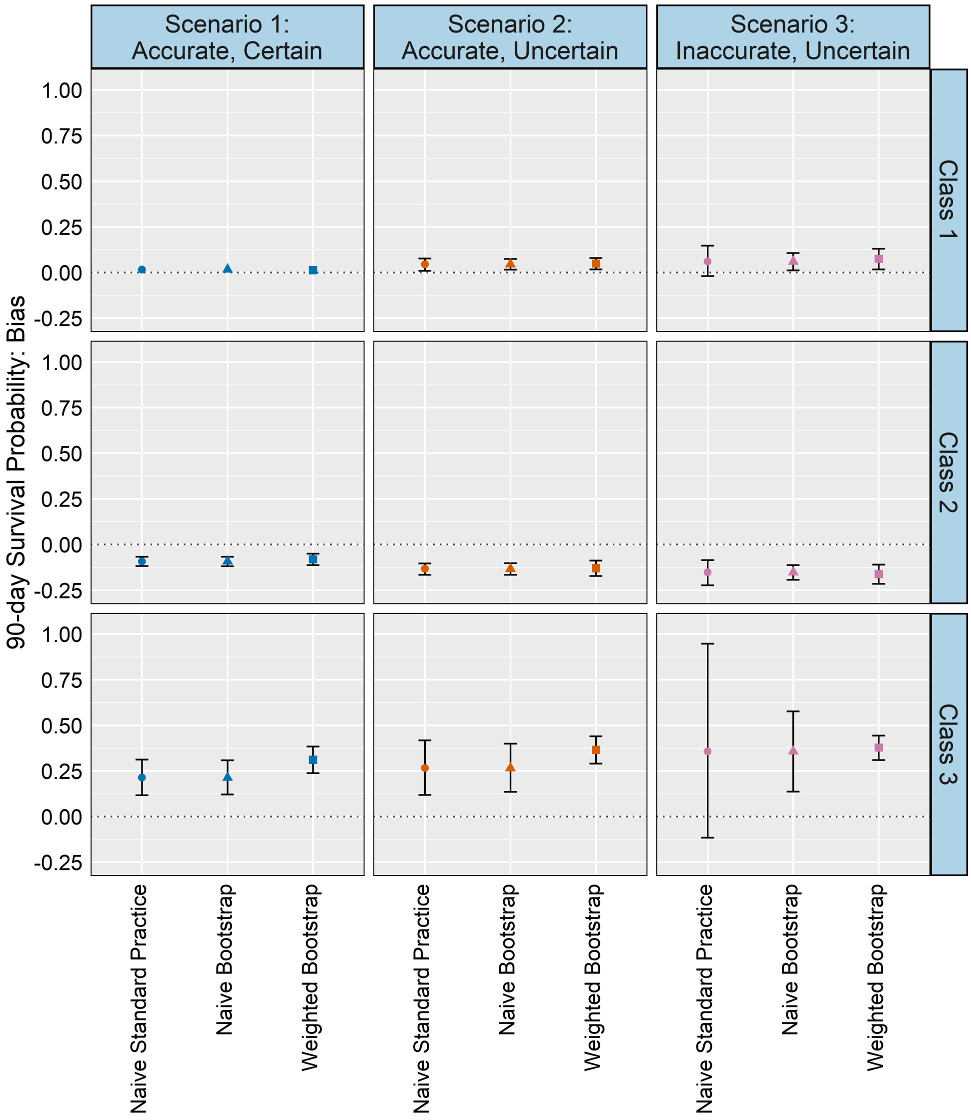

FIGURE B1 Simulation study 90-day survival probability bias.

(*For visual clarity, 95% confidence intervals less than 0.05 are not displayed.*)

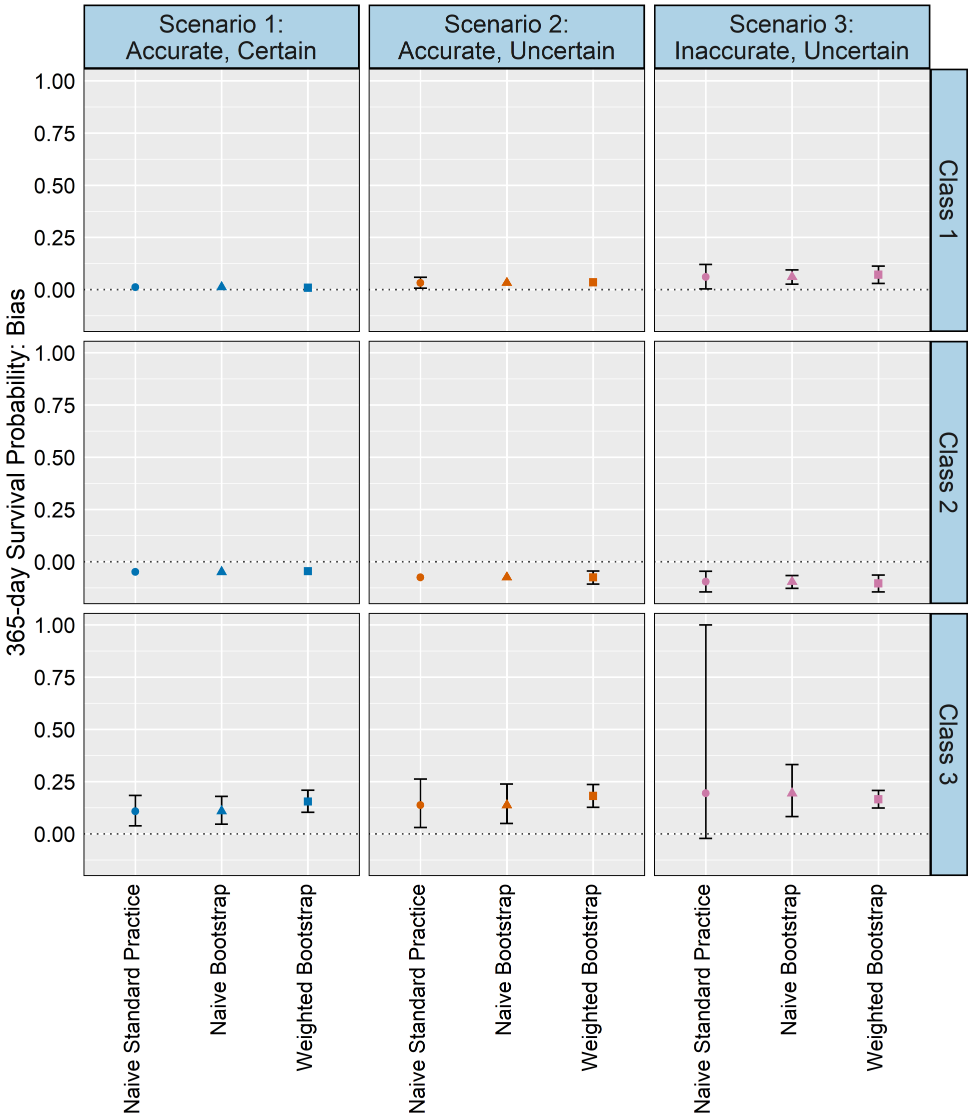

FIGURE B2 Simulation study 365-day survival probability bias.

(*For visual clarity, 95% confidence intervals less than 0.05 are not displayed.*)

C DATA ANALYSIS RESULTS

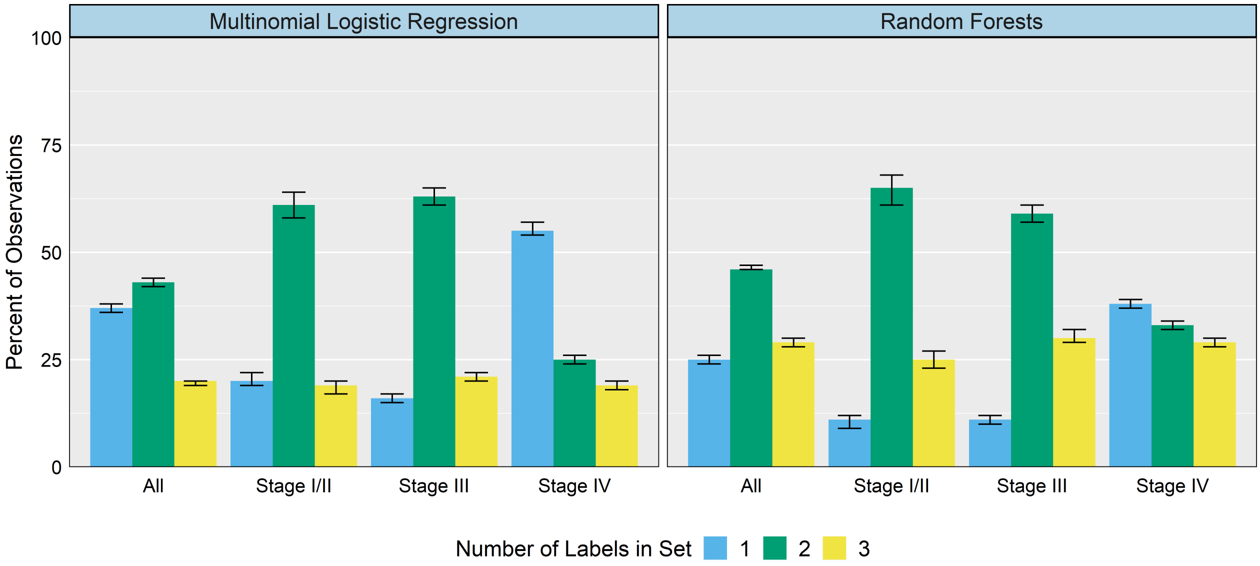

FIGURE C3 Data analysis label ambiguity: Share of sample by number of assigned labels in label set.

(*Results for additional algorithms omitted due to similarity with multinomial logistic regression. Error bars represent percentile-based 95% confidence intervals.*)

TABLE C15 Bootstrap-based coverage.

Algorithm	Stage I/II	Stage III	Stage IV
Weighted Labeling			
Elastic Net	0.89	0.90	0.90
	(0.88, 0.90)	(0.89, 0.90)	(0.89, 0.90)
Generalized Additive Regression	0.90	0.90	0.89
	(0.89, 0.92)	(0.89, 0.91)	(0.89, 0.90)
Lasso	0.89	0.90	0.90
	(0.88, 0.90)	(0.89, 0.91)	(0.89, 0.90)
Multinomial Logistic	0.90	0.90	0.89
	(0.89, 0.91)	(0.90, 0.91)	(0.89, 0.90)
Random Forests	0.94	0.91	0.90
	(0.93, 0.95)	(0.91, 0.92)	(0.90, 0.91)
Ridge	0.90	0.90	0.90
	(0.89, 0.91)	(0.89, 0.90)	(0.89, 0.91)
Gradient Boosting	0.91	0.91	0.90
	(0.90, 0.92)	(0.90, 0.92)	(0.90, 0.91)
Naive Labeling			
Elastic Net	0.32	0.61	0.84
	(0.30, 0.34)	(0.60, 0.63)	(0.83, 0.85)
Generalized Additive Regression	0.32	0.63	0.83
	(0.30, 0.34)	(0.61, 0.64)	(0.83, 0.84)
Lasso	0.32	0.61	0.84
	(0.30, 0.34)	(0.60, 0.63)	(0.83, 0.85)
Multinomial Logistic	0.32	0.61	0.84
	(0.30, 0.34)	(0.60, 0.62)	(0.83, 0.85)
Random Forests	0.30	0.58	0.87
	(0.30, 0.34)	(0.59, 0.62)	(0.84, 0.86)
Ridge	0.31	0.61	0.85
	(0.29, 0.33)	(0.59, 0.62)	(0.84, 0.86)
Gradient Boosting	0.30	0.58	0.86
	(0.28, 0.32)	(0.60, 0.63)	(0.84, 0.85)

TABLE C16 Weighted labeling thresholds.

Algorithm	Stage I/II	Stage III	Stage IV
Elastic Net	0.09	0.23	0.34
Generalized Additive Regression	0.07	0.21	0.31
Lasso	0.09	0.23	0.35
Multinomial Logistic	0.07	0.21	0.34
Random Forests	0.00	0.07	0.40
Ridge	0.09	0.23	0.36
Gradient Boosting	0.11	0.21	0.40

TABLE C17 Classification accuracy.

**Algorithm**	**Average**	**Stage I/II**	**Stage III**	**Stage IV**
Naive standard practice				
Elastic Net	0.79	0.87	0.73	0.78
Generalized Additive Regression	0.79	0.87	0.73	0.78
Lasso	0.79	0.87	0.73	0.78
Multinomial Logistic	0.79	0.87	0.73	0.78
Random Forests	0.79	0.87	0.73	0.77
Ridge	0.79	0.87	0.73	0.78
Gradient Boosting	0.79	0.87	0.74	0.77
Naive bootstrap				
Elastic Net	0.79	0.87	0.73	0.78
	(0.79, 0.80)	(0.86, 0.87)	(0.72, 0.74)	(0.77, 0.78)
Generalized Additive Regression	0.79	0.87	0.73	0.78
	(0.79, 0.80)	(0.86, 0.87)	(0.72, 0.74)	(0.77, 0.78)
Lasso	0.79	0.87	0.73	0.78
	(0.79, 0.80)	(0.86, 0.87)	(0.72, 0.74)	(0.77, 0.78)
Multinomial Logistic	0.79	0.87	0.73	0.77
	(0.79, 0.80)	(0.86, 0.87)	(0.72, 0.73)	(0.77, 0.78)
Random Forests	0.79	0.87	0.74	0.77
	(0.79, 0.80)	(0.86, 0.87)	(0.73, 0.74)	(0.76, 0.78)
Ridge	0.79	0.87	0.73	0.78
	(0.79, 0.80)	(0.86, 0.88)	(0.72, 0.74)	(0.77, 0.78)
Gradient Boosting	0.79	0.87	0.73	0.76
	(0.78, 0.79)	(0.86, 0.87)	(0.73, 0.74)	(0.75, 0.77)
Weighted bootstrap				
Elastic Net	0.73	0.75	0.67	0.75
	(0.72, 0.73)	(0.75, 0.76)	(0.67, 0.68)	(0.75, 0.76)
Generalized Additive Regression	0.72	0.74	0.67	0.75
	(0.71, 0.72)	(0.73, 0.75)	(0.66, 0.67)	(0.74, 0.75)
Lasso	0.73	0.75	0.67	0.75
	(0.72, 0.73)	(0.75, 0.76)	(0.66, 0.68)	(0.75, 0.76)
Multinomial Logistic	0.72	0.74	0.67	0.74
	(0.71, 0.72)	(0.74, 0.75)	(0.66, 0.67)	(0.74, 0.75)
Random Forests	0.68	0.67	0.66	0.71
	(0.67, 0.68)	(0.66, 0.68)	(0.65, 0.67)	(0.70, 0.71)
Ridge	0.73	0.75	0.67	0.76
	(0.72, 0.73)	(0.74, 0.76)	(0.66, 0.68)	(0.75, 0.76)
Gradient Boosting	0.71	0.73	0.66	0.75
	(0.71, 0.72)	(0.72, 0.74)	(0.66, 0.67)	(0.74, 0.75)

TABLE C18 Classification sensitivity.

Algorithm	Average	Stage I/II	Stage III	Stage IV
Naive standard practice				
Elastic Net	0.59	0.32	0.61	0.84
Generalized Additive Regression	0.59	0.32	0.63	0.83
Lasso	0.59	0.32	0.61	0.84
Multinomial Logistic	0.59	0.32	0.61	0.84
Random Forests	0.58	0.31	0.58	0.87
Ridge	0.59	0.31	0.61	0.85
Gradient Boosting	0.58	0.30	0.58	0.86
Naive bootstrap				
Elastic Net	0.59	0.32	0.61	0.84
	(0.58, 0.60)	(0.30, 0.34)	(0.60, 0.63)	(0.83, 0.85)
Generalized Additive Regression	0.59	0.33	0.63	0.83
	(0.59, 0.60)	(0.31, 0.35)	(0.62, 0.65)	(0.82, 0.83)
Lasso	0.59	0.32	0.61	0.84
	(0.58, 0.60)	(0.30, 0.34)	(0.60, 0.63)	(0.83, 0.85)
Multinomial Logistic	0.59	0.32	0.62	0.83
	(0.58, 0.60)	(0.30, 0.34)	(0.60, 0.63)	(0.83, 0.84)
Random Forests	0.57	0.28	0.56	0.88
	(0.57, 0.58)	(0.26, 0.30)	(0.54, 0.57)	(0.88, 0.89)
Ridge	0.59	0.31	0.61	0.85
	(0.58, 0.60)	(0.29, 0.33)	(0.59, 0.62)	(0.84, 0.86)
Gradient Boosting	0.58	0.3	0.55	0.88
	(0.57, 0.58)	(0.28, 0.32)	(0.53, 0.56)	(0.87, 0.88)
Weighted bootstrap				
Elastic Net	0.55	0.49	0.46	0.70
	(0.54, 0.56)	(0.47, 0.51)	(0.44, 0.47)	(0.69, 0.71)
Generalized Additive Regression	0.54	0.49	0.45	0.68
	(0.53, 0.55)	(0.47, 0.51)	(0.44, 0.47)	(0.67, 0.69)
Lasso	0.55	0.49	0.46	0.70
	(0.54, 0.56)	(0.47, 0.51)	(0.44, 0.47)	(0.69, 0.71)
Multinomial Logistic	0.54	0.49	0.45	0.68
	(0.53, 0.55)	(0.47, 0.51)	(0.44, 0.47)	(0.67, 0.69)
Random Forests	0.50	0.46	0.44	0.59
	(0.49, 0.50)	(0.44, 0.48)	(0.42, 0.45)	(0.58, 0.60)
Ridge	0.55	0.48	0.45	0.70
	(0.54, 0.56)	(0.46, 0.50)	(0.44, 0.47)	(0.69, 0.71)
Gradient Boosting	0.52	0.42	0.44	0.69
	(0.51, 0.53)	(0.40, 0.44)	(0.43, 0.46)	(0.68, 0.70)

TABLE C19 Classification specificity.

Algorithm	Average	Stage I/II	Stage III	Stage IV
Naive standard practice				
Elastic Net	0.82	0.96	0.79	0.71
Generalized Additive Regression	0.82	0.96	0.78	0.72
Lasso	0.82	0.96	0.79	0.71
Multinomial Logistic	0.82	0.96	0.78	0.71
Random Forests	0.82	0.97	0.81	0.67
Ridge	0.82	0.97	0.79	0.70
Gradient Boosting	0.81	0.97	0.81	0.66
Naive bootstrap				
Elastic Net	0.82	0.96	0.79	0.71
	(0.82, 0.82)	(0.96, 0.97)	(0.78, 0.79)	(0.7, 0.72)
Generalized Additive Regression	0.82	0.96	0.78	0.72
	(0.82, 0.83)	(0.96, 0.97)	(0.77, 0.78)	(0.71, 0.73)
Lasso	0.82	0.96	0.79	0.71
	(0.82, 0.82)	(0.96, 0.97)	(0.78, 0.79)	(0.7, 0.72)
Multinomial Logistic	0.82	0.96	0.78	0.71
	(0.82, 0.82)	(0.96, 0.97)	(0.77, 0.79)	(0.7, 0.72)
Random Forests	0.81	0.97	0.82	0.64
	(0.81, 0.82)	(0.97, 0.97)	(0.82, 0.83)	(0.63, 0.65)
Ridge	0.82	0.97	0.79	0.70
	(0.82, 0.82)	(0.96, 0.97)	(0.78, 0.8)	(0.69, 0.71)
Gradient Boosting	0.81	0.97	0.83	0.64
	(0.81, 0.81)	(0.96, 0.97)	(0.82, 0.83)	(0.62, 0.65)
Weighted bootstrap				
Elastic Net	0.80	0.80	0.78	0.81
	(0.79, 0.80)	(0.79, 0.81)	(0.77, 0.79)	(0.8, 0.82)
Generalized Additive Regression	0.79	0.79	0.77	0.82
	(0.79, 0.80)	(0.78, 0.79)	(0.76, 0.78)	(0.81, 0.82)
Lasso	0.80	0.80	0.78	0.81
	(0.79, 0.80)	(0.79, 0.80)	(0.77, 0.79)	(0.80, 0.82)
Multinomial Logistic	0.79	0.79	0.77	0.81
	(0.79, 0.80)	(0.78, 0.79)	(0.76, 0.78)	(0.81, 0.82)
Random Forests	0.77	0.71	0.77	0.83
	(0.77, 0.78)	(0.70, 0.72)	(0.76, 0.78)	(0.82, 0.84)
Ridge	0.80	0.80	0.78	0.81
	(0.79, 0.80)	(0.79, 0.80)	(0.77, 0.79)	(0.80, 0.82)
Gradient Boosting	0.79	0.78	0.77	0.80
	(0.78, 0.79)	(0.78, 0.79)	(0.76, 0.78)	(0.80, 0.81)

TABLE C20 Classification positive predictive value.

Algorithm	Average	Stage I/II	Stage III	Stage IV
Naive standard practice				
Elastic Net	0.65	0.61	0.59	0.76
Generalized Additive Regression	0.65	0.61	0.59	0.76
Lasso	0.65	0.61	0.59	0.76
Multinomial Logistic	0.65	0.61	0.59	0.76
Random Forests	0.66	0.63	0.60	0.74
Ridge	0.65	0.62	0.59	0.75
Gradient Boosting	0.65	0.62	0.61	0.73
Naive bootstrap				
Elastic Net	0.65	0.61	0.59	0.76
	(0.64, 0.66)	(0.59, 0.64)	(0.57, 0.60)	(0.75, 0.76)
Generalized Additive Regression	0.65	0.60	0.58	0.76
	(0.64, 0.66)	(0.57, 0.63)	(0.57, 0.60)	(0.76, 0.77)
Lasso	0.65	0.61	0.59	0.76
	(0.64, 0.66)	(0.58, 0.64)	(0.57, 0.60)	(0.75, 0.77)
Multinomial Logistic	0.65	0.60	0.59	0.76
	(0.64, 0.66)	(0.57, 0.63)	(0.57, 0.60)	(0.75, 0.77)
Random Forests	0.66	0.63	0.61	0.73
	(0.64, 0.67)	(0.60, 0.66)	(0.60, 0.63)	(0.72, 0.74)
Ridge	0.65	0.62	0.59	0.75
	(0.64, 0.67)	(0.59, 0.65)	(0.58, 0.60)	(0.74, 0.76)
Gradient Boosting	0.65	0.62	0.61	0.72
	(0.64, 0.66)	(0.59, 0.65)	(0.60, 0.63)	(0.71, 0.73)
Weighted bootstrap				
Elastic Net	0.53	0.30	0.51	0.80
	(0.53, 0.54)	(0.28, 0.31)	(0.49, 0.52)	(0.79, 0.81)
Generalized Additive Regression	0.53	0.28	0.50	0.08
	(0.52, 0.53)	(0.27, 0.30)	(0.48, 0.51)	(0.79, 0.81)
Lasso	0.53	0.30	0.51	0.80
	(0.53, 0.54)	(0.28, 0.31)	(0.49, 0.52)	(0.79, 0.81)
Multinomial Logistic	0.53	0.29	0.50	0.80
	(0.52, 0.53)	(0.27, 0.30)	(0.48, 0.51)	(0.79, 0.81)
Random Forests	0.50	0.22	0.49	0.79
	(0.49, 0.51)	(0.20, 0.23)	(0.47, 0.50)	(0.78, 0.80)
Ridge	0.53	0.29	0.50	0.80
	(0.53, 0.54)	(0.28, 0.31)	(0.49, 0.52)	(0.79, 0.81)
Gradient Boosting	0.51	0.25	0.49	0.79
	(0.51, 0.52)	(0.24, 0.27)	(0.48, 0.51)	(0.78, 0.80)

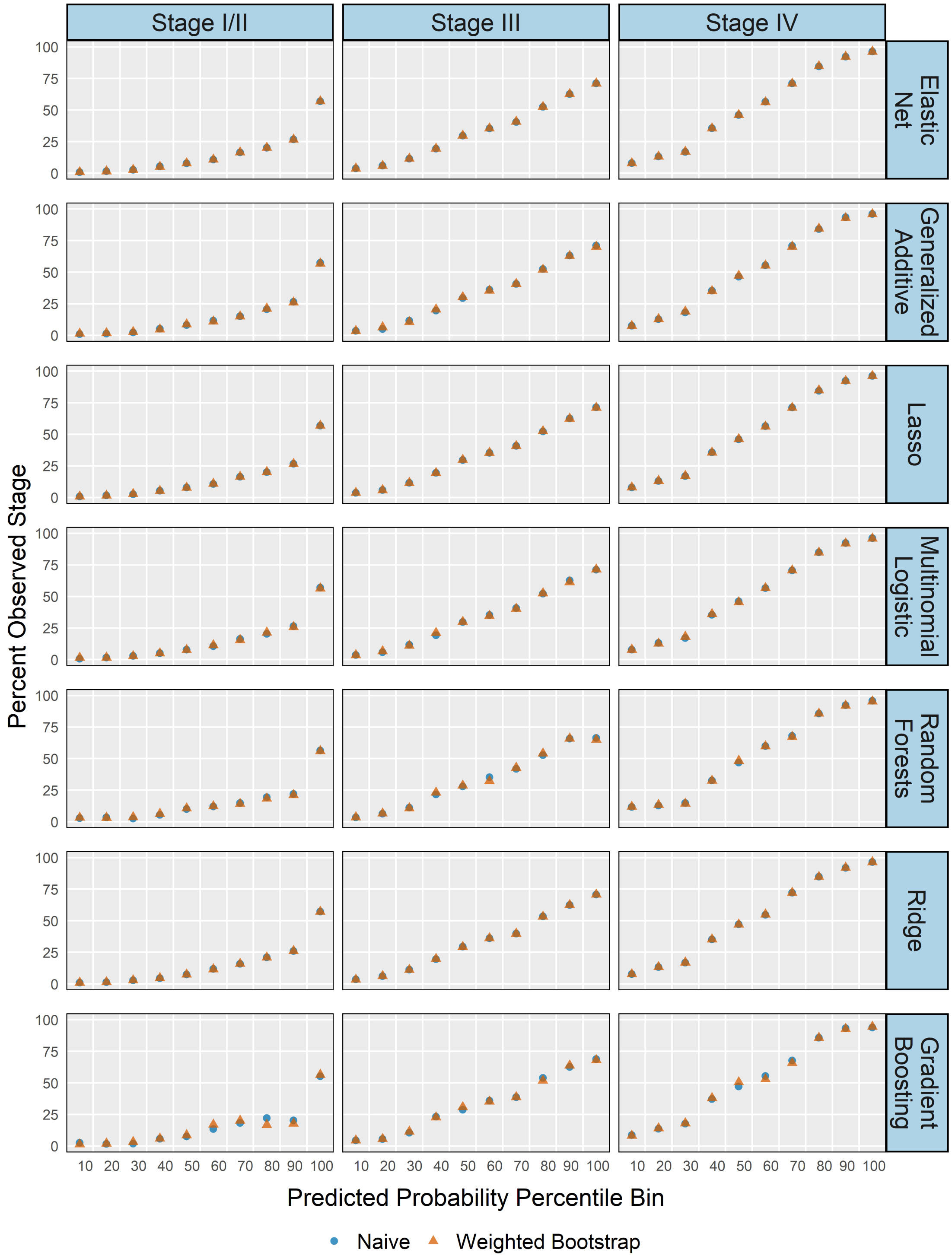

FIGURE C4 Data analysis: Observed stage by predicted probability.

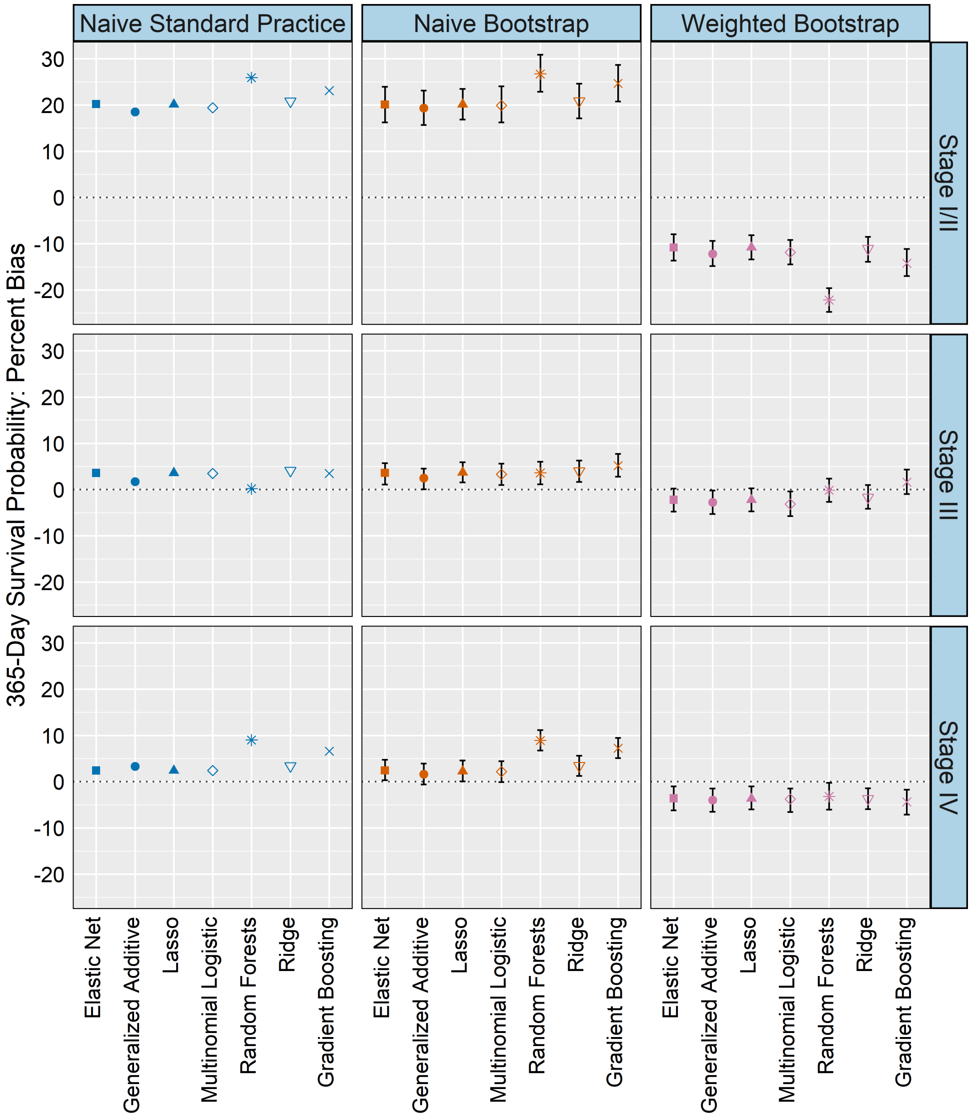

FIGURE C5 Data analysis: 365-day survival probability percent bias.

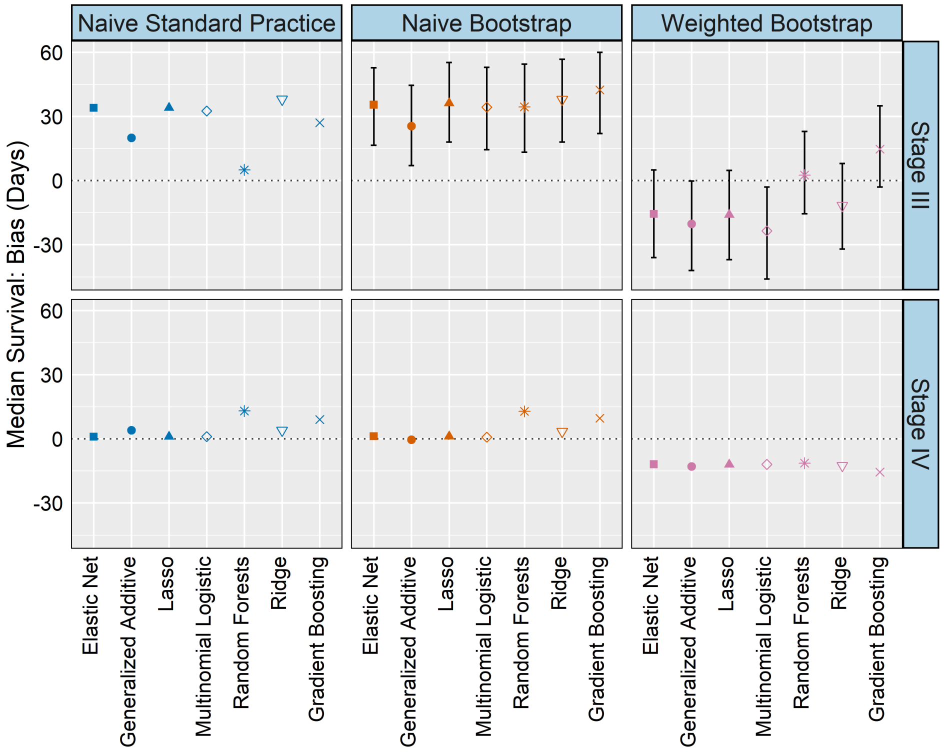

FIGURE C6 Data analysis: Median days survival bias.

(*For visual clarity, 95% confidence intervals less than 31 days are not displayed.*)

TABLE C21 Data analysis: Median days survival bias.

**Algorithm**	**Stage III**	**Stage IV**
Naive standard practice		
Elastic Net	34	1
Generalized Additive	20	4
Lasso	34	1
Multinomial Logistic	32	1
Random Forests	5	13
Ridge	38	4
Gradient Boosting	27	9
Naive bootstrap		
Elastic Net	35	1
	(16, 53)	(-3, 6)
Generalized Additive	25	0
	(7, 45)	(-5, 4)
Lasso	36	1
	(18, 55)	(-3, 6)
Multinomial Logistic	34	1
	(14, 53)	(-4, 5)
Random Forests	34	13
	(13, 54)	(8, 17)
Ridge	38	3
	(18, 57)	(0, 7)
Gradient Boosting	42	10
	(22, 60)	(5, 14)
Weighted bootstrap		
Elastic Net	-16	-12
	(-36, 5)	(-17, -7)
Generalized Additive	-20	-13
	(-42, 0)	(-19, -7)
Lasso	-16	-12
	(-37, 5)	(-17, -7)
Multinomial Logistic	-24	-12
	(-46, -3)	(-18, -7)
Random Forests	3	-11
	(-16, 23)	(-17, -6)
Ridge	-12	-13
	(-32, 8)	(-18, -8)
Gradient Boosting	15	-16
	(-3, 35)	(-21, -11)

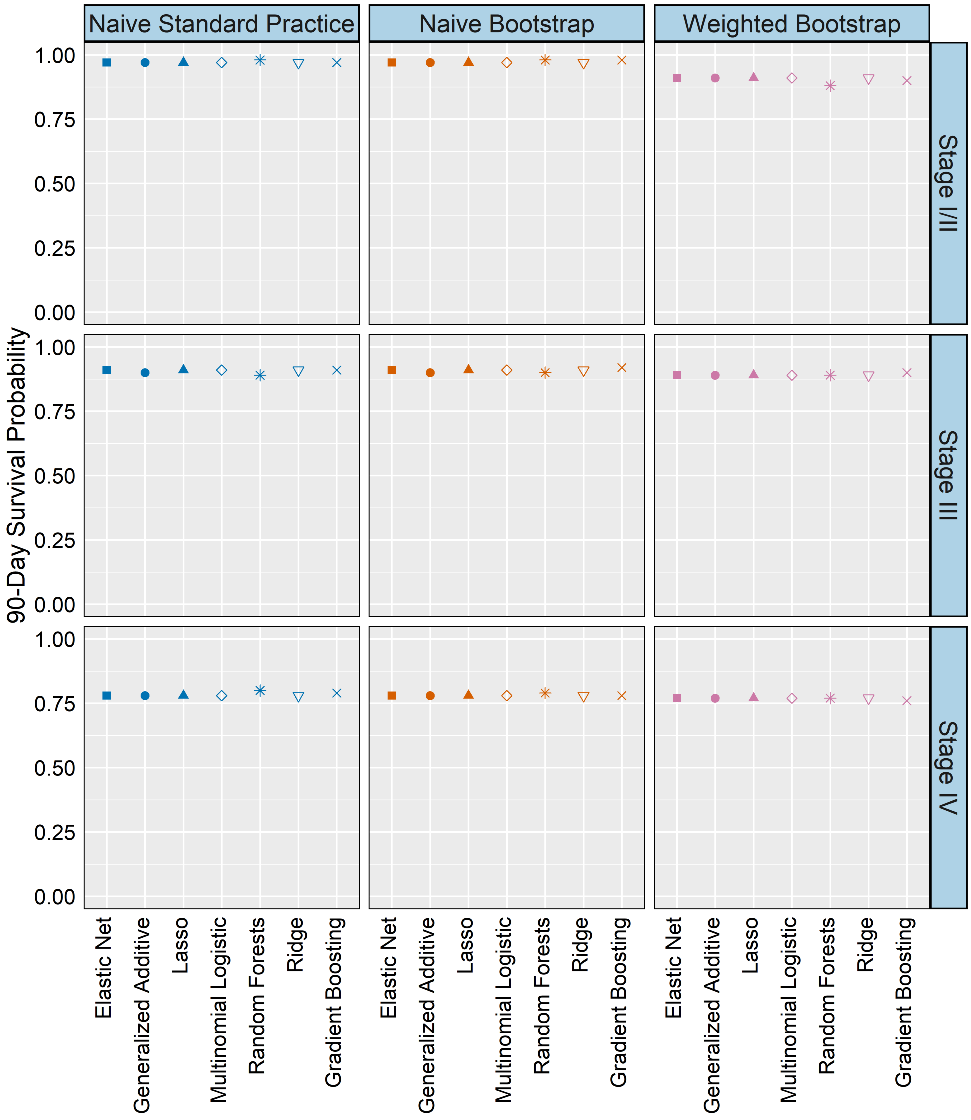

FIGURE C7 Data analysis: 90-day survival by predicted stage.

(*For visual clarity, 95% confidence intervals less than 0.05 are not displayed.*)

TABLE C22 Data analysis: 90-day survival by predicted stage.

Algorithm	Stage I/II	Stage III	Stage IV
Naive standard practice			
Elastic Net	0.97	0.91	0.78
	(0.96, 0.98)	(0.9, 0.92)	(0.77, 0.79)
Generalized Additive	0.97	0.9	0.78
	(0.95, 0.98)	(0.89, 0.91)	(0.78, 0.79)
Lasso	0.97	0.91	0.78
	(0.96, 0.98)	(0.9, 0.92)	(0.77, 0.79)
Multinomial Logistic	0.97	0.91	0.78
	(0.96, 0.98)	(0.9, 0.92)	(0.77, 0.79)
Random Forests	0.98	0.89	0.8
	(0.97, 0.99)	(0.88, 0.9)	(0.79, 0.8)
Ridge	0.97	0.91	0.78
	(0.96, 0.98)	(0.90, 0.92)	(0.77, 0.79)
Gradient Boosting	0.97	0.91	0.79
	(0.96, 0.98)	(0.90, 0.92)	(0.78, 0.8)
Naive bootstrap			
Elastic Net	0.97	0.91	0.78
	(0.96, 0.98)	(0.90, 0.92)	(0.77, 0.79)
Generalized Additive	0.97	0.9	0.78
	(0.96, 0.98)	(0.89, 0.91)	(0.77, 0.79)
Lasso	0.97	0.91	0.78
	(0.96, 0.98)	(0.90, 0.91)	(0.77, 0.79)
Multinomial Logistic	0.97	0.91	0.78
	(0.96, 0.98)	(0.90, 0.91)	(0.77, 0.79)
Random Forests	0.98	0.9	0.79
	(0.97, 0.99)	(0.89, 0.91)	(0.79, 0.8)
Ridge	0.97	0.91	0.78
	(0.96, 0.98)	(0.9, 0.92)	(0.78, 0.79)
Gradient Boosting	0.98	0.92	0.78
	(0.97, 0.98)	(0.92, 0.93)	(0.78, 0.79)
Weighted bootstrap			
Elastic Net	0.91	0.89	0.77
	(0.90, 0.92)	(0.88, 0.90)	(0.76, 0.78)
Generalized Additive	0.91	0.89	0.77
	(0.90, 0.91)	(0.88, 0.90)	(0.76, 0.78)
Lasso	0.91	0.89	0.77
	(0.90, 0.92)	(0.88, 0.9)	(0.76, 0.78)
Multinomial Logistic	0.91	0.89	0.77
	(0.90, 0.92)	(0.88, 0.9)	(0.76, 0.78)
Random Forests	0.88	0.89	0.77
	(0.87, 0.89)	(0.88, 0.90)	(0.76, 0.78)
Ridge	0.91	0.89	0.77
	(0.90, 0.92)	(0.88, 0.90)	(0.76, 0.78)
Gradient Boosting	0.90	0.90	0.76
	(0.89, 0.91)	(0.89, 0.91)	(0.75, 0.78)

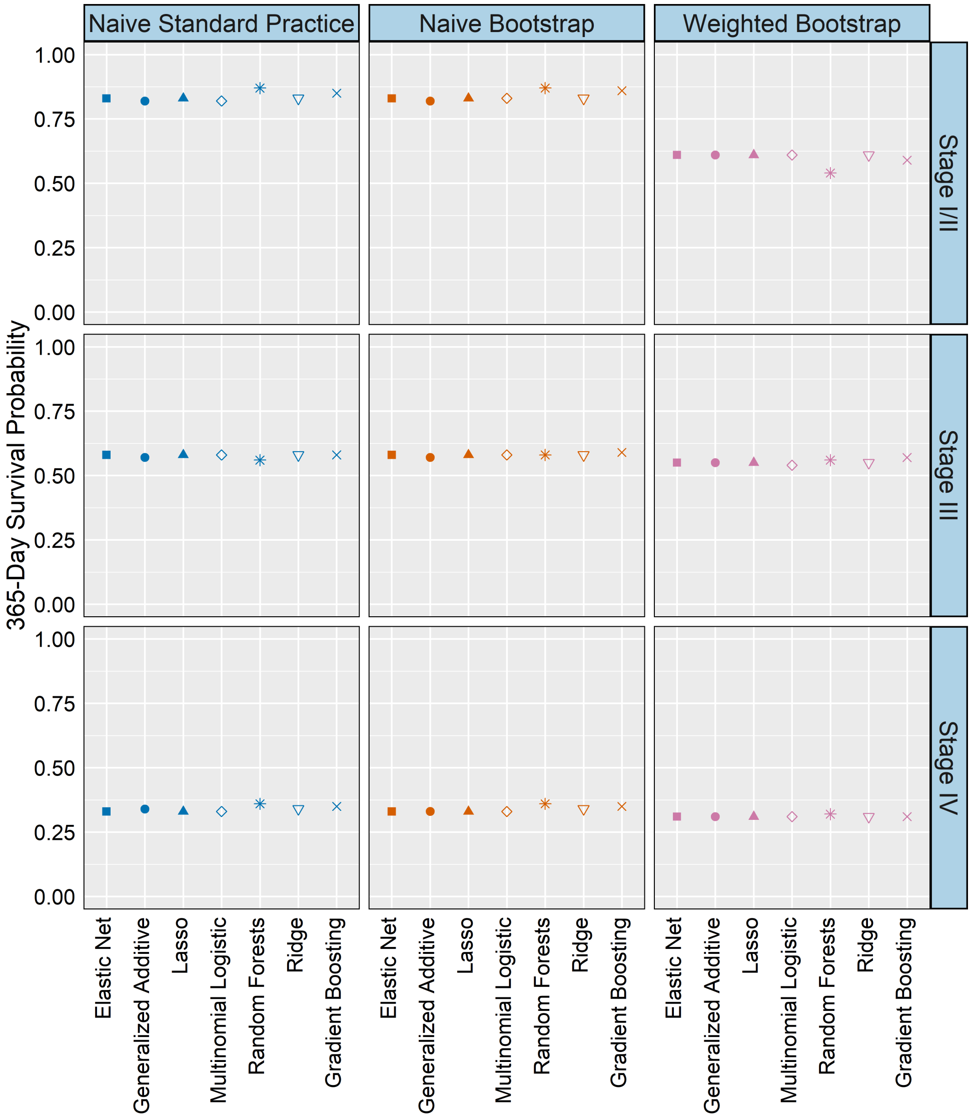

FIGURE C8 Data analysis: 365-day survival by predicted stage.

(*For visual clarity, 95% confidence intervals less than 0.05 are not displayed.*)

TABLE C23 Data analysis: 365-day survival by predicted stage.

Algorithm	Stage I/II	Stage III	Stage IV
Naive standard practice			
Elastic Net	0.83	0.58	0.33
	(0.81, 0.85)	(0.57, 0.59)	(0.32, 0.34)
Generalized Additive	0.82	0.57	0.34
	(0.79, 0.84)	(0.56, 0.58)	(0.33, 0.35)
Lasso	0.83	0.58	0.33
	(0.81, 0.85)	(0.57, 0.59)	(0.32, 0.34)
Multinomial Logistic	0.82	0.58	0.33
	(0.8, 0.84)	(0.57, 0.59)	(0.32, 0.34)
Random Forests	0.87	0.56	0.36
	(0.85, 0.89)	(0.55, 0.58)	(0.35, 0.37)
Ridge	0.83	0.58	0.34
	(0.81, 0.85)	(0.57, 0.6)	(0.33, 0.35)
Gradient Boosting	0.85	0.58	0.35
	(0.83, 0.87)	(0.57, 0.59)	(0.34, 0.36)
Naive bootstrap			
Elastic Net	0.83	0.58	0.33
	(0.8, 0.85)	(0.57, 0.59)	(0.32, 0.34)
Generalized Additive	0.82	0.57	0.33
	(0.8, 0.84)	(0.56, 0.59)	(0.32, 0.34)
Lasso	0.83	0.58	0.33
	(0.81, 0.85)	(0.57, 0.59)	(0.32, 0.34)
Multinomial Logistic	0.83	0.58	0.33
	(0.8, 0.85)	(0.56, 0.59)	(0.32, 0.34)
Random Forests	0.87	0.58	0.36
	(0.85, 0.89)	(0.57, 0.59)	(0.35, 0.37)
Ridge	0.83	0.58	0.34
	(0.81, 0.85)	(0.57, 0.6)	(0.33, 0.35)
Gradient Boosting	0.86	0.59	0.35
	(0.84, 0.88)	(0.58, 0.6)	(0.34, 0.36)
Weighted bootstrap			
Elastic Net	0.61	0.55	0.31
	(0.6, 0.63)	(0.53, 0.56)	(0.30, 0.33)
Generalized Additive	0.61	0.55	0.31
	(0.59, 0.62)	(0.53, 0.56)	(0.30, 0.33)
Lasso	0.61	0.55	0.31
	(0.6, 0.63)	(0.53, 0.56)	(0.30, 0.33)
Multinomial Logistic	0.61	0.54	0.31
	(0.59, 0.62)	(0.53, 0.56)	(0.30, 0.32)
Random Forests	0.54	0.56	0.32
	(0.52, 0.55)	(0.54, 0.57)	(0.30, 0.33)
Ridge	0.61	0.55	0.31
	(0.60, 0.63)	(0.54, 0.57)	(0.30, 0.32)
Gradient Boosting	0.59	0.57	0.31
	(0.58, 0.61)	(0.56, 0.58)	(0.30, 0.32)

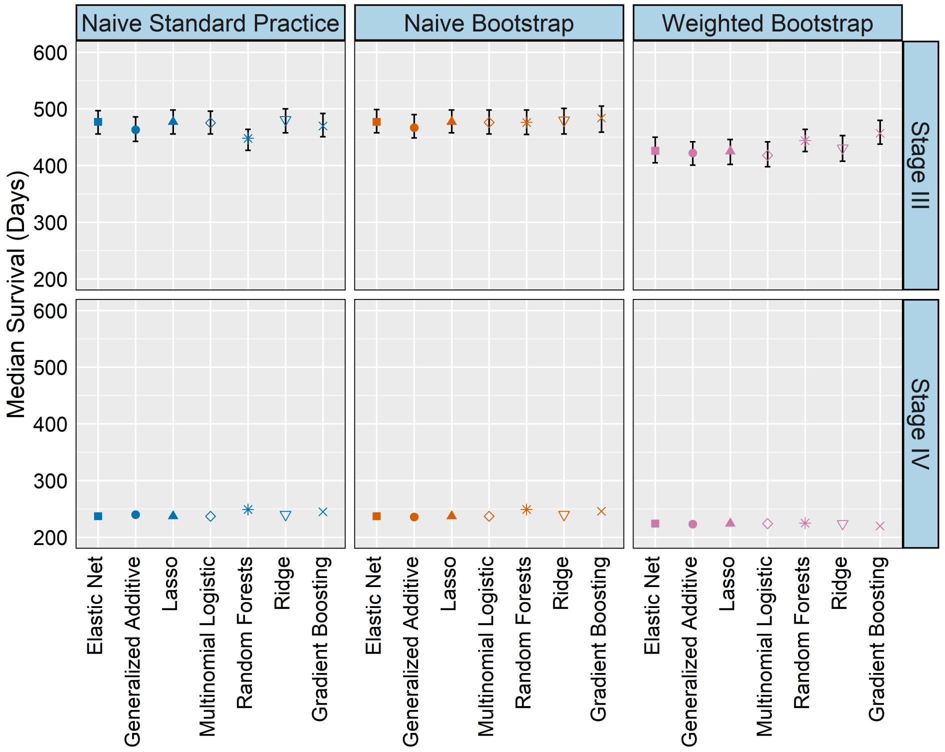

FIGURE C9 Data analysis: Median survival by predicted stage.

(*For visual clarity, 95% confidence intervals less than 31 days are not displayed.*)

TABLE C24 Data analysis: Median days survival by predicted stage.

Algorithm	Stage III	Stage IV
Naive standard practice		
Elastic Net	477	237
	(456, 497)	(231, 245)
Generalized Additive	463	240
	(443, 486)	(232, 246)
Lasso	477	237
	(456, 498)	(231, 245)
Multinomial Logistic	475.5	237
	(456, 496)	(230, 244)
Random Forests	448	249
	(427, 464)	(244, 255)
Ridge	481	240
	(458, 500)	(232, 246)
Gradient Boosting	470	245
	(451, 492)	(238, 250)
Naive bootstrap		
Elastic Net	477	237
	(458, 499)	(231, 245)
Generalized Additive	467	236
	(449, 490)	(230, 242)
Lasso	477	237
	(458, 498)	(231, 245)
Multinomial Logistic	476	237
	(456, 498)	(231, 244)
Random Forests	476	249
	(455, 498)	(244, 254)
Ridge	480	240
	(456, 501)	(233, 247)
Gradient Boosting	484	246
	(459, 505)	(239, 251)
Weighted bootstrap		
Elastic Net	426	224
	(405, 450)	(217, 231)
Generalized Additive	422	223
	(401, 442)	(216, 230)
Lasso	425	224
	(402, 446)	(218, 231)
Multinomial Logistic	418	224
	(398, 442)	(218, 231)
Random Forests	444	225
	(425, 464)	(218, 232)
Ridge	431	224
	(408, 453)	(218, 230)
Gradient Boosting	457	220
	(438, 480)	(214, 227)
